# Food insecurity in high-risk rural communities before and during the COVID-19 pandemic

**DOI:** 10.1016/j.heliyon.2024.e31354

**Published:** 2024-05-17

**Authors:** Carolina Quintero Arias, Melissa Rony, Erica Jensen, Rahi Patel, Stasha O'Callaghan, Christian A. Koziatek, Kelly M. Doran, Rebecca Anthopolos, Lorna E. Thorpe, Brian Elbel, David C. Lee

**Affiliations:** a Ronald O. Perelman Department of Emergency Medicine, NYU School of Medicine, New York, NY, 10016, USA; b Touro College of Osteopathic Medicine, New York, NY, 10027, USA; c Northeast Ohio Medical University, Rootstown, OH, 44272, USA; d Department of Population Health, NYU School of Medicine, New York, NY, 10016, USA

**Keywords:** Rural health, Food insecurity, Health disparities, Global pandemic, COVID-19

## Abstract

**Objective:**

To perform a geospatial analysis of food insecurity in a rural county known to have poor health outcomes and assess the effect of the COVID-19 pandemic.

**Methods:**

In 2020, we mailed a comprehensive cross-sectional survey to all households in Sullivan County, a rural county with the second-worst health outcomes among all counties in New York State. Surveys of households included validated food insecurity screening questions. Questions were asked in reference to 2019, prior to the pandemic, and for 2020, in the first year of the pandemic. Respondents also responded to demographic questions. Raking adjustments were performed using age, sex, race/ethnicity, and health insurance strata to mitigate non-response bias. To identify significant hotspots of food insecurity within the county, we also performed geospatial analysis.

**Findings:**

From the 28,284 households surveyed, 20% of households responded. Of 4725 survey respondents, 26% of households reported experiencing food insecurity in 2019, and in 2020, this proportion increased to 35%. In 2020, 58% of Black and Hispanic households reported experiencing food insecurity. Food insecurity in 2020 was also present in 58% of unmarried households with children and in 64% of households insured by Medicaid. The geospatial analyses revealed that hotspots of food insecurity were primarily located in or near more urban areas of the rural county.

**Conclusions:**

Our countywide health survey in a high-risk rural county identified significant increases of food insecurity in the first year of the COVID-19 pandemic, despite national statistics reporting a stable rate. Responses to future crises should include targeted interventions to bolster food security among vulnerable rural populations.

## 1 Introduction

The COVID-19 pandemic resulted in significant loss of life, disability, and additional strain on an healthcare system already operating under substantial stress [1]. Simultaneously, the pandemic has caused economic disruption for many households in the United States, which negatively impacted both physical and mental health [2]. At the intersection of health and the economy lies food insecurity [3]. Food insecurity is defined as “a lack of consistent access to enough food for an active, healthy life,” and it disproportionately affects seniors, children, minorities, and rural Americans [4]. In past economic downturns, such as the recession of 2008, there were sharp increases in food insecurity [5]. Therefore, it might also be expected that food insecurity had increased due to the COVID-19 pandemic.

In a survey conducted by the United States Department of Agriculture (USDA), national rates of food insecurity appeared to remain stable from 2019 to 2020, affecting approximately 10.5% of households in both years [6]. Substantial expansions in government aid during the pandemic have been cited as the explanation for this finding [7]. The U.S. Government sent out three rounds of direct relief payments during the COVID-19 pandemic that each started in March 2020 (up to $1200 per adult and $500 per dependent child), December 2020 (up to $600 per adult or dependent child), and March 2021 (up to $1400 per adult or dependent adult/child) [8,9]. Despite the overall stability in food insecurity reported, not all groups had been equally protected during the pandemic [10]. The same USDA report also found widening disparities in food insecurity, potentially by race and ethnicity, due to the disproportionate impact of the pandemic on minority populations, barriers in accessing available aid, and fewer existing assets to provide protection in times of economic instability [11].

However, there have been other studies of food insecurity during the pandemic that have come to different conclusions using alternative measurement approaches. For instance, projections from Feeding America suggest that rates of food insecurity increased nationally from 10.9 % in 2019 to 15.6 % in 2020 [12]. Notably, their methodology is based on modelled estimates that incorporate key factors such as unemployment and poverty rates.

The goal of this study was to perform a comprehensive assessment of pre-pandemic and early pandemic food insecurity in Sullivan County, New York. As a rural county with the second-poorest health ranking among all counties in New York State, it may have been especially vulnerable to the stressors of the pandemic [13]. We used this approach as it would not be based on modelled estimates, but instead directly queried the same survey participants as to whether they experienced food insecurity in 2019 and 2020. By using a mailed survey, we were also able to perform a detailed assessment of food insecurity within a given county with address-level geographic precision.

## 2 Methods

### 2.1 Study design

We administered a comprehensive cross-sectional survey of all households in Sullivan County, NY (See Appendix for the complete survey used). The survey included two validated food insecurity screening questions [14]. The questions were asked in reference to 2019, prior to the pandemic, and during the first year of the pandemic in 2020. Respondents also responded to questions about demographics and household characteristics. Raking adjustments were performed using age, sex, race/ethnicity, and health insurance strata To help mitigate non-response bias [15]. We also identified significant clusters of food insecurity within the county using geospatial analysis.

### 2.2 Mailed health surveys

To perform our cross-sectional survey in Sullivan County, we obtained a comprehensive list of all households from the Marketing Systems Group (Horsham, PA). The list was obtained in October 2020 and contained all residential households including those with a post office box address. Excluding seasonal and vacant housing, the final list contained a total of 28,284 households, similar to the estimated 28,184 households in the 2019 American Community Survey (ACS) estimates for Sullivan County. To maximize coverage across the county, we mailed a health survey in January 2021 to all households on the final list. The health survey first included questions confirming residence within Sullivan County and then asked a brief series of health and demographic questions. Survey respondents were offered a gift card of $10 for participation. A stamped, pre-addressed, return envelope was also included in the mailing. Responses were returned to the Sullivan County Public Health Services and then sent to the NYU School of Medicine for data entry and analysis.

### 2.3 Main study outcomes

Our main study outcome was self-reported food insecurity at two time points: before the COVID-19 pandemic in 2019 and during the first year of the pandemic in 2020. We used two previously validated screening questions to identify food insecurity. Participants were asked whether, “In 2019 before the pandemic, we worried about whether our food would run out before we got money to buy more” with possible answer choices “often true, sometimes true, and never true.” The next question asked whether the statement was also true in 2020 and had the same answer choices as the previous question. Participants were then asked whether, “In 2019 before the pandemic, the food we bought just didn't last and we didn't have enough money to get more” followed by asking whether the statement was also true in 2020 using the same answer choices (often true, sometimes true, and never true). Food insecurity was defined as an answer of “often true” or “sometimes true” to either question in the respective year referenced [14].

### 2.4 Demographic factors

Survey respondents were also asked to fill out household and demographic questions. These questions included their age, sex, race/ethnicity, health insurance, marital status, and the number of adults and children living in their household. Given the large number of seasonal residents in Sullivan County, we also asked whether survey respondents were full-time or part-time residents in the county. For all analyses, we excluded part-time residents so that our study population would only include full-time residents of the county. To compare survey respondents to the adult population in Sullivan County and to account for non-response bias, we obtained Census population demographic estimates for these demographic and household characteristics from the 2019 ACS.

### 2.5 Statistical analysis

We first performed descriptive analyses of health survey respondents with ACS-derived county population demographics in terms of age, sex, race/ethnicity, health insurance, and household characteristics. We examined these characteristics for health survey respondents overall and for those who endorsed any level food insecurity in 2019 and in 2020. For the analysis of our main study outcomes, we performed raking using age, sex, race/ethnicity, and health insurance strata to mitigate non-response bias [16]. The ACS-derived county population demographic estimates for the county were used as a reference for the target population. Approximately 2 % of survey respondents were missing data for one of the raking variables. Given this small proportion, we restricted this analysis to complete cases. The race/ethnicity categories for Asian and Other were combined to avoid small cell sizes. Raking estimates were also compared to naïve estimates to ensure that there was not any abnormal distortion of the results.

We also assessed for statistically significant changes in food insecurity between 2019 and 2020, and whether these changes in food insecurity were driven by age, sex, race/ethnicity, insurance status, or household type. Using generalized estimating equations to account for correlation between food insecurity responses from the same participant, we fit a marginal logistic regression model of food insecurity as a function of all of the demographic variables, an indicator for year, and the interaction terms between each demographic variable and year [17]. To test the null hypothesis of no difference in food insecurity by year, we used a Wald test. For demographic variables with more than two categories, the multivariate Wald test was used. A p-value of 0.05 was used to identify statistically significant differences.

### 2.6 Geographic analysis

We also performed clustering analysis within the county to identify significant hot and cold spots of food insecurity. Mailing addresses were geocoded to pinpoint the exact location of residence. Survey respondents with a post office box address were excluded from this analysis given the lack of a precise location. The outcome of food insecurity was analyzed using Getis-Ord Gi* hotspot analysis [18]. We used the K-nearest neighbors to model spatial proximity as there was significant variation in distance between nearest neighborhoods within this rural county. The number of nearest neighbors was specified as 100 neighbors with sensitivity analyses at 50 and 200 neighbors. A false discovery rate correction was applied to account for multiple testing and spatial dependency. The resultant z-scores and p-values determined whether a given point and its K-nearest neighbors represented a hotspot of food insecurity.

Statistical analyses were performed using Stata 16.1 (Statacorp; College Station, TX, 2019) and R 4.1.2 (2021-11-01). Geographic analysis and mapping were performed using ArcGIS Pro 2.8.3 (ESRI; Redlands, CA, 2021).

## 3 Results

### 3.1 Mailed survey responses

Of the 28,284 mailed health surveys, 2706 (10 %) were returned to sender. The five most common reasons for these returns were vacant address, unable to forward, no mail receptacle, insufficient address, and addressee unknown. Of the remaining 25,578 surveys, we received 5230 responses (20 %), which was similar to our expectation based on our prior experience performing a one-time mailed health survey in this rural region [19]. Twenty-eight of the returned surveys were partially completed and could not be used for the analysis due to missing data on food insecurity. We excluded an additional 468 survey respondents who reported being part-time residents of the county and nine who lived outside the borders of Sullivan County. Our final analytic sample included a total of 4725 full-time residents of Sullivan County.

### 3.2 Population characteristics

As shown in Table 1, compared to Census estimates for the adult population in Sullivan County, survey respondents were generally older (i.e., 10 % in the study population versus 5 % in the Census estimates that were 80 years or older), more frequently female (57 % in the study population versus 49 % in the Census estimates), more frequently non-Hispanic White (85 % in the study population versus 70 % in the Census estimates), more likely to be insured by Medicare (41 % in the study population versus 20 % in the Census estimates), and more frequently married without children (37 % in the study population versus 28 % in the Census estimates).

**Table 1:** Study population characteristics.

Population Characteristics	Census Estimates	Health Survey Respondents	2019 Food Insecurity	2020 Food Insecurity
Total Adults	59,174	4725	1052	1404
Age				
18 to 39	19,138 (32 %)	566 (12 %)	156 (15 %)	214 (15 %)
40 to 59	20,666 (35 %)	1348 (29 %)	367 (35 %)	484 (35 %)
60 to 79	16,680 (28 %)	2306 (49 %)	447 (43 %)	594 (43 %)
80 or older	2690 (5 %)	477 (10 %)	72 (7 %)	98 (7 %)
Sex				
Male	30,331 (51 %)	2039 (43 %)	399 (38 %)	551 (39 %)
Female	28,843 (49 %)	2658 (57 %)	647 (62 %)	847 (61 %)
Race/Ethnicity				
White	43,103 (70 %)	3965 (85 %)	741 (71 %)	1014 (73 %)
Black	4939 (8 %)	199 (4 %)	93 (9 %)	107 (8 %)
Hispanic	9582 (16 %)	367 (8 %)	168 (16 %)	207 (15 %)
Asian	1355 (2 %)	56 (1 %)	13 (1 %)	21 (1 %)
Other	2807 (5 %)	93 (2 %)	26 (3 %)	39 (3 %)
Health Insurance				
Private	27,619 (49 %)	1805 (38 %)	244 (23 %)	399 (29 %)
Medicare	11,097 (20 %)	1909 (41 %)	320 (31 %)	443 (32 %)
Medicaid	10,331 (18 %)	758 (16 %)	418 (40 %)	464 (33 %)
Self-Pay	7155 (13 %)	238 (5 %)	64 (6 %)	91 (7 %)
Households				
Single, no kids	12,690 (45 %)	1966 (42 %)	555 (53 %)	712 (51 %)
Single, with kids	2779 (10 %)	348 (7 %)	161 (15 %)	188 (14 %)
Married, no kids	7994 (28 %)	1716 (37 %)	180 (17 %)	297 (21 %)
Married, with kids	4721 (17 %)	653 (14 %)	145 (14 %)	194 (14 %)

### 3.3 Food insecurity before the pandemic in 2019

**Fig. 1:**
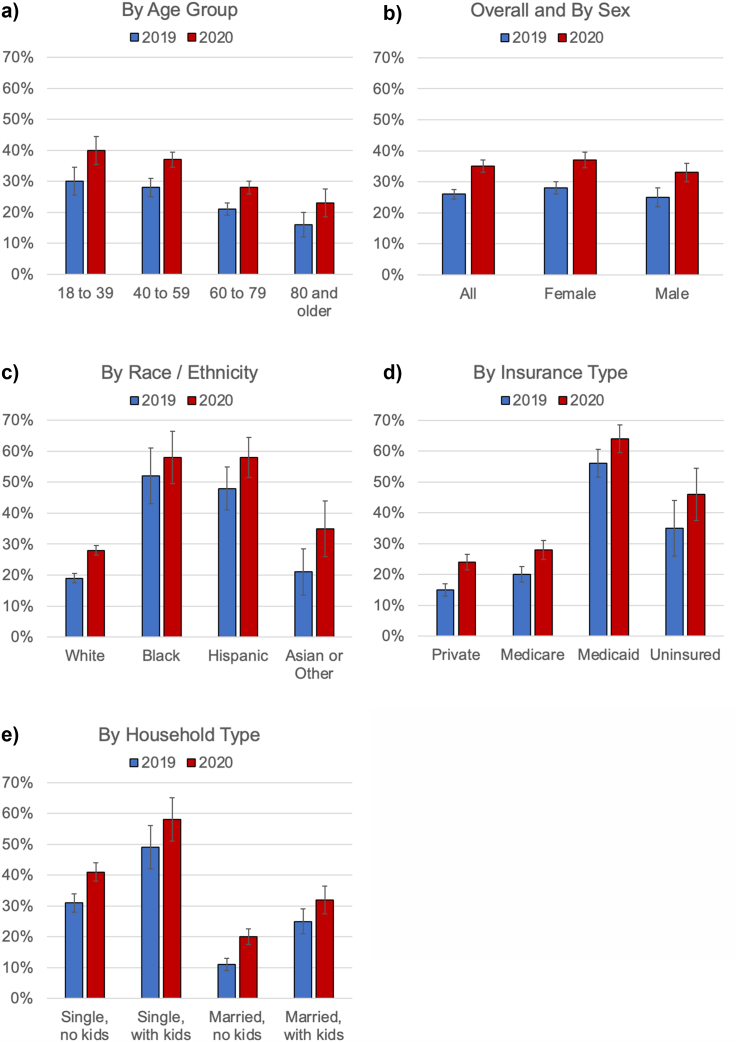
Food Insecurity by Demographics in Sullivan County for 2019 and 2020. Notes: Rates of Food Insecurity Stratified by a) age, b) sex, c) race/ethnicity, d) insurance type, and e) household type.

In 2019, the raking weighted estimate of food insecurity was 26 % in Sullivan County. Food insecurity was higher among younger age groups, with 30 % of 18- to 39-year-olds and 28 % of 40- to 59-year-olds reporting food insecurity, compared to 21 % of 60- to 79-year-olds and 16 % of respondents 80 years and older. We found large disparities in food insecurity by race and ethnicity, with 52 % of Black and 48 % of Hispanic respondents reporting food insecurity compared to 21 % of Asian and Other respondents and 19 % of White respondents. In addition, we noted significant disparities in food insecurity by health insurance status and household type, with 56 % of respondents insured by Medicaid reporting food insecurity and 49 % of single respondents with children reporting food insecurity (Fig. 1).

### 3.4 Food insecurity during the pandemic in 2020

Respondents reported experiencing food insecurity more frequently during the first year of the pandemic, increasing from 26 % in 2019 to 35 % in 2020. This increase in the prevalence of food insecurity was statistically significantly (p < 0.001). While there were considerable baseline disparities in food insecurity in 2019, our analysis suggested that food insecurity increased proportionally among all subgroups studied by age, sex, race/ethnicity, health insurance, and household type. We did not find that the increase in food insecurity was driven by any specific sociodemographic factor: age (p = 0.54), sex (p = 0.79), race/ethnicity (p = 0.73), insurance status (p = 0.14), or household type (p = 0.35). These findings are in line with distribution of population characteristics among health survey respondents reporting food insecurity in 2019 and 2020 (Table 1).

### 3.5 Geospatial clustering of food insecurity

**Fig. 2:**
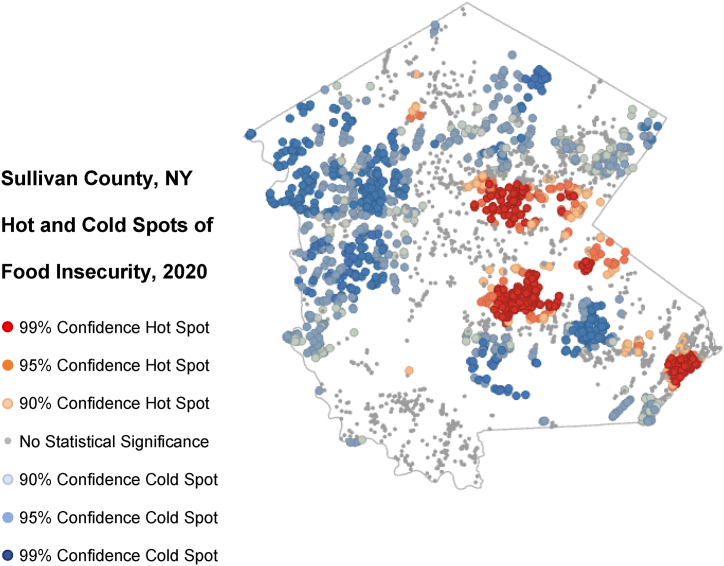
Geographic clustering of food insecurity within Sullivan County in 2020.

In our geospatial analysis, we assessed whether there was any statistically significant clustering of food insecurity in 2020 using the closest 100 neighbors for each geocoded residential location. The map of Sullivan County in Fig. 2 demonstrates the hot and cold spots with 90 %, 95 %, and 99 % confidence. The two largest hotspots of food insecurity were concentrated in the two more densely populated towns, which by Census definitions, are known as urbanized clusters. Our sensitivity analysis using the closest 50 and 200 neighbors demonstrated a similar clustering of these hot and cold spots, differing slightly only in the relative size of areas identified.

## 4 Discussion

In 2019, our survey showed that rural Sullivan County, New York already experienced levels of food insecurity that were more than double the levels of the national average. In 2020, we found a large increase in reported food insecurity among Sullivan County residents during the first year of the COVID-19 pandemic, affecting more than one in three households. Although USDA data suggests that national food insecurity rates have stayed relatively stable despite the pandemic, these averages may not reflect sharp increases experienced in certain high-risk regions of the country, such as Sullivan County, which may have had a distinctly different experience of the COVID-19 pandemic [6]. In Sullivan County, several factors such as poverty, low income, and unemployment may have accounted for the stark increase in food insecurity during the pandemic. These predisposing factors would be consistent with other studies that also reported the pandemic's significant impact of food insecurity among low-income individuals and those who lost jobs or wages during the pandemic [20,21].

In comparison to the USDA report, our study results are more in line with other studies by national organizations like Feeding America that have suggested that food insecurity increased nationally between 2019 and 2020. Though the stability of estimates from USDA reports may not apply specifically to food insecurity in Sullivan County, we also note an important limitation in the USDA report that might have affected their own estimates. During the pandemic, USDA survey response rates dropped dramatically from 83 % pre-pandemic to a low of 65 % in their repeated cross-sectional surveys [6]. If non-response was higher among those more likely to report food insecurity, then the levels of food insecurity reported by the USDA in 2020 may have been significantly underestimated and may have missed the experience of disadvantaged respondents.

In terms of the demographic groups most affected by food insecurity in Sullivan County, we found that Black and Hispanic residents reported food insecurity at over double the rate of non-Hispanic White residents. Some studies have suggested that racial and ethnic disparities in food insecurity have widened disproportionately among urban minorities during the COVID-19 pandemic [22]. However, our data suggested that food insecurity did not necessarily increase disproportionately for minorities in Sullivan County from 2019 to 2020. Rather, disparities already observed for Black and Hispanic residents in 2019 increased comparably to other groups during the first year of the pandemic.

In analyzing our results by age group, seniors are often considered a subgroup that is particularly at-risk for food insecurity [23]. However, in our study of Sullivan County, we found much higher baseline rates of food insecurity among younger adults. These rates also increased proportionally across age groups due to the COVID-19 pandemic. These younger age groups may have been more affected by the economic hardships and job loss experienced during the pandemic compared to older age groups, which may have qualitatively experienced a different set of hardships during the pandemic [24]. Notably, baseline rates of food insecurity were 3.7 times higher among residents insured by Medicaid compared to residents with private health insurance and were also 1.6 times higher among residents insured by Medicaid compared to residents without health insurance. These findings are in line with prior studies that suggest that Medicaid recipients specifically are at high risk for food insecurity as they often face not only economic difficulties, but also higher rates of chronic diseases such as diabetes. These health problems can lead to high healthcare expenses, which further complicates paying for basic expenses such as food [25,26]. Evaluating food insecurity by insurance status between 2019 and 2020, increases during the pandemic affected residents across all health insurance subgroups.

Our study also showed that baseline food insecurity was especially high for single households with children. This is consistent with data from the USDA, which demonstrates that single-mother households account for some of the highest levels of food insecurity [6]. During the COVID-19 pandemic, households with children not only had to deal with the economic hardships of the pandemic, but also faced additional difficulties as school closures, which not only meant the challenge of additional childcare, but loss of access to school lunches [27]. While there were programs to attempt to replace the loss of these resources, deployment of pickup school lunch programs can be more difficult in rural areas due to much farther distance between home and school. At the same time, our data did suggest that all household subgroups experienced increases in food insecurity between 2019 and 2020, suggesting that existing disparities in food insecurity persisted, without significant widening nor narrowing, during the pandemic.

The high rates of food insecurity that we identified in Sullivan County at baseline and the increases during the pandemic are concerning. Food insecurity has a direct impact on physical and mental health [28,29]. Higher rates of food insecurity are correlated with higher body-mass index and elevated rates of chronic disease [30]. Individuals who are food insecure have much higher rates of hypertension, diabetes, and heart disease [31]. Along with these overall physical effects of food insecurity, there are other important population-specific health issues that can arise from food insecurity [32]. Research has documented significant mental health effects of food insecurity in single household families with children. For example, food insecure mothers have double the rates of mental health issues compared to food-secure mothers [33]. In children, food insecurity can lead to physical and mental developmental issues as well as behavioral problems [33].

In our geospatial analysis, we were surprised to find that food insecurity demonstrated statistically significant clustering in specific regions of the county. Most of these hotspots were located in the more urban areas of the rural county, with two of them directly located in urban clusters within Sullivan County. While this clustering does not mean an absence of remote rural residents facing substantial food insecurity, it does suggest several possibilities. First, poor access to food in rural regions has largely been described as food deserts, meaning long distances to supermarkets or other food sources [34,35]. However, many of these studies have been performed with poor geographic specifications and the within-county clustering of food insecurity suggests the opposite trend as these hotspots are centered in the areas of Sullivan County that are closest to the available food sources. In addition, it suggests that addressing food insecurity can capitalize on this clustering by targeting resources to those more densely populated areas where food insecurity may be particularly high.

### 4.1 Strengths and limitations

Our study was a detailed, countywide health survey of a single rural county in New York State known to have particularly poor health outcomes. Particular strengths our study include performing a geographically precise assessment of within-county food insecurity, which allowed us to evaluate food insecurity by specific areas within the rural county and by key demographic groups. Our findings may not be generalizable to other rural areas of New York State or the rest of the country. In addition, our survey was a single cross-sectional survey with questions about status in two consecutive years. As discussed, this approach may have led to recall bias that might have inflated our estimates of food insecurity, although it negates also concerns about repeated surveys when response rates differ dramatically between years as was seen in the USDA reports. Finally, while we used raking to adjust for measured characteristics of survey respondents to yield estimates more representative of the county, it is possible that unmeasured characteristics differ among respondents and non-respondents, which may have a significant impact on estimates obtained in our analyses. Furthermore, the Census estimates from the ACS may also be limited by the relatively few sampling points within the county, which may have affected our adjusted estimates of food insecurity.

## 5 Conclusions

In considering effective policy responses to the pandemic and addressing baseline disparities in food insecurity, it is important to carefully consider how these programs should be designed. It may be that we need to be more directed and targeted in our approach to dealing with the economic hardships of health and economic crises. In our study, we found particular groups, including Black and Hispanic households, those insured by Medicaid, and single households with children, face disproportionately high baseline levels of food insecurity. Effective policy interventions to reduce food insecurity may need to consider income levels for directed assistance and allocate additional resources to target other specific demographic features, such as Black and Hispanic households, single household with children, and those insured by Medicaid. This would not only alleviate economic hardships due to crises such as the recent COVID-19 pandemic but could also lead to more positive overall health outcomes amongst these high-risk groups.

## Data availability

The data that support the findings of this study are available from the corresponding author, Dr. David C. Lee, upon reasonable request. Researchers interested in obtaining the data can contact the corresponding authors at the following email address to make the request: David.Lee@nyulangone.org.

## Ethics statement

This study was reviewed and approved by the Institutional Review Board at the NYU School of Medicine with the approval number: i19-01920. A waiver of written informed consent was granted for the analysis of the survey results given the minimal risks of the study; however, all participants were made aware that their participation was voluntary and that their individual responses would remain confidential. A copy of this language is in the survey document that can be found in the Appendix.

## Funding

This study was funded by grant R01-DK124400 from the 10.13039/100000062National Institute of Diabetes and Digestive and Kidney Diseases, which is focused on understanding the environmental and geographic risk factors for diabetes in rural areas of the country. The funder had no role in the design, conduct, drafting of the manuscript, or decision to submit the study for publication.

## CRediT authorship contribution statement

**Carolina Quintero Arias:** Writing – original draft, Resources, Project administration, Data curation. **Melissa Rony:** Writing – review & editing, Resources, Project administration, Data curation. **Erica Jensen:** Writing – review & editing, Resources, Data curation. **Rahi Patel:** Writing – review & editing, Resources, Data curation. **Stasha O'Callaghan:** Writing – review & editing, Resources, Data curation. **Christian A. Koziatek:** Writing – review & editing, Resources, Methodology, Investigation. **Kelly M. Doran:** Writing – review & editing, Resources, Methodology, Investigation. **Rebecca Anthopolos:** Writing – review & editing, Resources, Methodology, Investigation, Formal analysis. **Lorna E. Thorpe:** Writing – review & editing, Resources, Methodology, Investigation, Conceptualization. **Brian Elbel:** Writing – review & editing, Resources, Methodology, Investigation, Conceptualization. **David C. Lee:** Writing – original draft, Visualization, Validation, Supervision, Resources, Project administration, Methodology, Investigation, Funding acquisition, Formal analysis, Data curation, Conceptualization.

## Declaration of competing interest

The authors declare that they have no known competing financial interests or personal relationships that could have appeared to influence the work reported in this paper.
