# Supplementary material for: Food insecurity in high-risk rural communities before and during the COVID-19 pandemic
Source: Heliyon. 2024 May 17;10(10):e31354. doi: 10.1016/j.heliyon.2024.e31354 (PMC11130676; doi:10.1016/j.heliyon.2024.e31354)
Supplement: Multimedia component 1 [file mmc1.docx]

**
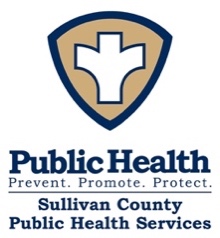

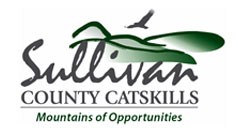
Sullivan County Public Health Services**

50 Community Lane, PO Box 590

Liberty, New York 12754

**2021 Sullivan County Health Survey**

The Sullivan County Public Health Services and NYU School of Medicine have partnered to perform a health survey sponsored by the National Institutes of Health to better understand the health of residents in Sullivan County. The survey takes 5 to 10 minutes and you will receive a $10 gift card by mail for your time. It is completely voluntary and your responses will be kept confidential. No information will be collected or used to identify you or any other individuals taking the survey. You must be a Sullivan County resident to be eligible. Choose only one adult from your household to answer. **If you received two surveys, please only return one survey.**

1. How many years have you lived in Sullivan County? ___________ Years

2. How many months of each year do you live in Sullivan County? _____ of 12 Months

Have you EVER been told by a doctor or other health professional that you have / had:

3. Hypertension or High Blood Pressure Yes No

4. Hyperlipidemia or High Cholesterol Yes No

5. Coronary Heart Disease, Angina, or Heart Attack Yes No

6. Stroke Yes No

7. Prediabetes or Borderline Diabetes Yes No

8. Gestational or Pregnancy Related Diabetes Yes No

9. Diabetes Yes, Type 1 Yes, Type 2 Yes, Unknown Type No

10. Do you have a mother, father, sister, or brother with diabetes? Yes No

Approximately 150 mins of moderate intensity or 75 mins of vigorous intensity exercise each week

11. Are you physically active? Yes No

12. How tall are you without shoes? _______ Feet and _______ Inches

13. How much do you weigh? _______ Pounds

14. On average, how many hours of sleep do you get in a 24-hour period? _______ Hours

15. How long has it been since you last saw a doctor or health care professional about your health?

Past Year Last 2 Years Last 3 Years Last 5 Years Last 10 Years 10 Years or More

16. What kinds of health insurance or health care coverage do you have? Circle all that apply.

Private or Medicare Medicaid Self-Pay Other Insurance No Coverage

Commercial

*Please turn the page over for additional questions.*

*The following questions will help us understand some of the basic needs of your household:*

17. In **2019** before the pandemic, we worried about whether our food would run out before

we got money to buy more: Often True Sometimes True Never True

18. In **2020**, the above statement was: Often True Sometimes True Never True

19. In **2019** before the pandemic, the food we bought just didn’t last and we didn’t have

enough money to get more: Often True Sometimes True Never True

20. In **2020**, the above statement was: Often True Sometimes True Never True

*In order to provide an accurate assessment of health in Sullivan County, we need to ask the following basic demographic information about you and your household:*

21. How many adults live in your household including yourself? _______ Adults

How many children under the age of 18 live in your household? _______ Children

22. What is your current marital status? Single Married Widowed Divorced Separated

23. What is your age? 18 to 29 30 to 39 40 to 49 50 to 59 60 to 69 70 to 79 80 or older

24. Are you? Male Female Other

25. Are you Hispanic, Latino, or Spanish in origin? Yes No

26. Which of these is your race? White Black Asian Other

*Would you like your gift card sent to the same address we used to mail this survey?*

Yes No, please mail it to _________________________________________


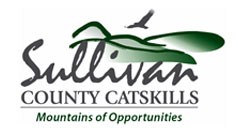
**
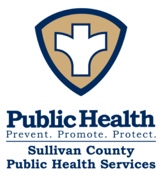
**

Survey Number ________________________________________
